# Case of Cholera at Cape May

**Published:** 1849-11

**Authors:** Francis West

**Affiliations:** Philadelphia


*Case of Cholera at Cape May.* Reported by FRANCIS WEST, M. D., Philadelphia.

The following case of cholera maligna fell under the care of the writer at Cape May, during the bathing season in August last. The number of visitors there at the time was about 1000.

The occurrence of isolated or single cases of this disease, in localities of more than ordinary salubrity, and the non-extension of it under these circumstances to other individuals, would seem to be *matters of fact*, of considerable interest and importance in their bearing upon the etiology and mode of propagation of this remarkable disease. There is no disorder, perhaps, which, in regard to these two points, has more perfectly illustrated the opposite experience of medical men, gathered under apparently precisely similar circumstances, than the epidemic in question; the history of its rise and progress, at one time and in some particular place, appearing directly to contradict, in many points, the accounts which are given of it on other occasions and in different situations.

M. S., a robust and perfectly healthy Irish girl, aged about 23 years, had visited the Island as a domestic in the family of Mr. M—— of this city. When they left Philadelphia, the cholera was quite on the decline, if, indeed, it had not almost entirely disappeared as an epidemic. It was not until exactly one week after her arrival at Cape May, that she was seized with the attack of cholera which in a few hours terminated her life. As far as I could learn, she had been troubled with diarrhœa only a day or two previously, but during this period, as well as throughout her whole stay upon the Island, she had been indulging very freely in all kinds of rich food, besides vegetables and fruits, to which she had not before been accustomed. At noon on the day of her attack, she had taken her bath as usual in the sea, along with her mistress, and had made no complaint whatever of indisposition; her dinner too was taken as usual. About five o'clock in the afternoon, I was requested by Mr. M——, quite incidentally, during a visit paid to the house where the family were staying, "to look at his servant girl, who did not appear to be very well, and had just gone to lie down." I found that she had had some diarrhœa and vomiting, for which a few doses of "cholera mixture," brought from the city, had been given to her, but her condition had not awakened the slightest solicitude in the family. Upon examination of the patient, I was immediately struck with the extreme feebleness and smallness of her pulse, which seemed to be inexplicable by the amount of diarrhœa and vomiting. There was no opportunity at this time of observing the character of the discharges. Her skin was cool, and her tongue also did not present the usual warmth. There was *something*, however, about her, which, taken in connexion with the state of her pulse, skin, and tongue, made me fear that the attack was not one of ordinary cholera morbus, and on parting with Mr. M——, I said to him, "if this girl was in Philadelphia, or under the influence of the cholera poison, I should say that she had the disease." I prescribed some camphor mixture, composed of Aq. Camphoræ ℥iij. Spir. Lavand. comp. ℥j. Spir. Ammoniæ Aromat. ʒij. Tinct. Opii. f.ʒss., together with a large sinapism over the abdomen, and a hot mustard pediluvium. In the course of an hour I repeated my visit, having left her with serious misgivings in regard to the nature of the case. During my absence she had had no repetition of the diarrhœa, and had only once vomited. Upon my return, however, I found no improvement of the pulse, but, on the contrary, a further diminution of its size and strength, with marked increase of the superficial coolness, and, added to all this, a sensible sinking of the eyes, whispering voice, shrivelling of the fingers, commencing blueness of the limbs, face and body, cramps in the extremities, &c.; in a word, all the symptoms of incipient collapse. My worst fears being now confirmed, I at once requested, and as quickly obtained, the valuable counsel of Professor Darrach, of this city, who happened to be staying in the same house. He agreed with me in the opinion that it was a decided case of cholera maligna in its worst form, and suggested that we should give a fair trial to the practice of exhibiting calomel in grain doses every five minutes, with ten drops of laudanum in a teaspoonful of brandy. These were accordingly given, and at the same time sinapisms were freely applied to various portions of the body. Within a short time afterwards, the pulse seemed to rally a little, but no other improvement was manifested; on the contrary, all the symptoms soon became aggravated; immense dejections of real rice-water fluid occurred about 10 o'clock, P. M., and continued, at short intervals, with the effect of rapidly exhausting the system. At about 12 o'clock, P. M., the state of collapse was perfect, and she soon sank into a moribund condition, during all which time her mind remained perfectly clear. She died about 5 o'clock, A. M., on the following morning. Dr. D. and myself remained with her from the time when we first met until 2 o'clock the next morning. Several persons besides the family were present, and assisted us with their kind offices. Her body was removed on the following day to this city for interment. I remained for some time after her death, but no other case of the disease made its appearance, either in the house where she died, or at any other point upon the island.

This case of course presents no features of special interest, except in regard to the two points referred to in the early part of this communication. The entire absence of any previous or subsequent cases of the disease upon the island, would seem to prove, beyond question, that this solitary case was not contracted there, and if so, that the " semina morbi " had remained latent in the patient's system during the entire week which had elapsed between the time of her departure from Philadelphia and that of her arrival at Cape May; their full development not having been susceptible of control, even by the salubrious atmosphere of the sea-shore. This fact goes to prove, further, that the cases of cholera which have occasionally shown themselves on ship-board at different periods after the departure of the vessel from an infected place, may really have been cont acted on shore, thus doing away the necessity, presumed to exist from their deferred appearance, of supposing that the vessel had struck at sea upon a vein of cholera atmosphere. The opinion of the *directly* contagious character of cholera would also seem to be entirely disproved, by the non-extension of the disease among the individuals in attendance upon this patient. Such a case, indeed, as it appears to us, is, of all others, best calculated to afford satisfactory proof upon this particular point. Had the condition of the patient been such as to favor *infection* in others who were about her, it is more than probable that the disease would have spread there, as elsewhere, under the influence of similar agencies.

